# Stimulating at the right time: phase-specific deep brain stimulation

**DOI:** 10.1093/brain/aww286

**Published:** 2016-12-22

**Authors:** Hayriye Cagnan, David Pedrosa, Simon Little, Alek Pogosyan, Binith Cheeran, Tipu Aziz, Alexander Green, James Fitzgerald, Thomas Foltynie, Patricia Limousin, Ludvic Zrinzo, Marwan Hariz, Karl J Friston, Timothy Denison, Peter Brown

**Affiliations:** 1 1 Institute of Neurology, University College London, London, UK; 2 2 Nuffield Department of Clinical Neurosciences, University of Oxford, Oxford, UK; 3 3 Medical Research Council Brain Network Dynamics Unit, University of Oxford, Oxford, UK; 4 4 Medtronic Neuromodulation, Minneapolis, USA

**Keywords:** essential tremor, dystonic tremor, synchrony, ventrolateral thalamus, closed-loop stimulation

## Abstract

**See Moll and Engel (doi:10.1093/aww308) for a scientific commentary on this article**.

Brain regions dynamically engage and disengage with one another to execute everyday actions from movement to decision making. Pathologies such as Parkinson’s disease and tremor emerge when brain regions controlling movement cannot readily decouple, compromising motor function. Here, we propose a novel stimulation strategy that selectively regulates neural synchrony through phase-specific stimulation. We demonstrate for the first time the therapeutic potential of such a stimulation strategy for the treatment of patients with pathological tremor. Symptom suppression is achieved by delivering stimulation to the ventrolateral thalamus, timed according to the patient’s tremor rhythm. Sustained locking of deep brain stimulation to a particular phase of tremor afforded clinically significant tremor relief (up to 87% tremor suppression) in selected patients with essential tremor despite delivering less than half the energy of conventional high frequency stimulation. Phase-specific stimulation efficacy depended on the resonant characteristics of the underlying tremor network. Selective regulation of neural synchrony through phase-locked stimulation has the potential to both increase the efficiency of therapy and to minimize stimulation-induced side effects.

## Teaser

During pathologies like tremor, neural populations become locked into temporal configurations that reinforce neural synchrony to the point that motor function is compromised. Cagnan *et al.* present a novel stimulation approach to selectively control neural synchrony, and demonstrate that deep brain stimulation can be precisely timed to disrupt disease pathophysiology.

## Introduction

The temporal relationship between neural activities is one of the most fundamental neural properties that determines the degree of information exchange between distributed brain regions, and dictates short and long-term plasticity (Hebb, 2002; Fries, 2005). Neural populations engaged in rhythmic activity frequently shift between configurations that promote enhancement of neural synchrony and those that suppress it, encoding vital information for behavior (Womelsdorf *et al.*, 2007; Cagnan *et al.*, 2015*b*). During pathologies such as tremor, oscillating neural populations in the cerebello-thalamo-cortical network become locked into temporal configurations that reinforce neural synchrony to the point that motor function is compromised (Schnitzler *et al.*, 2009). Theoretical and experimental studies suggest that neural oscillators can be moved to and from such critical temporal relationships by carefully timed pulses of stimulation (Smeal *et al.*, 2010; Akam *et al.*, 2012; Wilson and Moehlis, 2014; Zlotnik *et al.*, 2016). Here, we experimentally test this principal, and present a novel approach to selectively control neural synchrony through phase-interference, and demonstrate that stimulation based therapies such as deep brain stimulation (DBS) can be precisely timed to ‘decouple’ the neural network to selectively reduce local and circuit-level synchrony.

DBS is a widely used surgical intervention, used in the treatment of debilitating neurological disorders such as advanced Parkinson’s disease, essential tremor, and dystonia (Benabid *et al.*, 1991; Rodriguez-Oroz *et al.*, 2005; Okun, 2012; Miocinovic *et al.*, 2013; Ostrem *et al.*, 2014), and its efficacy is being explored with investigational research for disorders such as obsessive-compulsive disorder (Kohl *et al.*, 2014), Tourette’s syndrome (Schrock *et al.*, 2015) and epilepsy (Vonck *et al.*, 2012). DBS modulates local neural activity with brief electrical pulses delivered via chronically implanted electrodes in the subcortical brain regions that are involved in disease pathophysiology. Continuous high frequency stimulation (130–180 Hz) of subcortical motor nuclei has proven to be highly effective in suppressing Parkinson’s disease motor symptoms, and tremor observed in essential and dystonic tremor. However, the disruptive effects of high frequency stimulation are not necessarily specific for the neural signals driving disease symptoms, and associated disruption of physiological activity may explain some stimulation-induced side effects such as dysarthria, reduced verbal fluency, and impairments in balance and gait (Zhang *et al.*, 2009; Baizabal-Carvallo *et al.*, 2013). While lowering the amount of energy delivered reduces stimulation-induced side effects, to date low frequency stimulation (<20 Hz) has not been clinically effective in suppressing disease symptoms, and in certain instances could increase symptom severity (Hassler *et al.*, 1960; Constantoyannis *et al.*, 2004; Pedrosa *et al.*, 2014).

**Figure 1:**
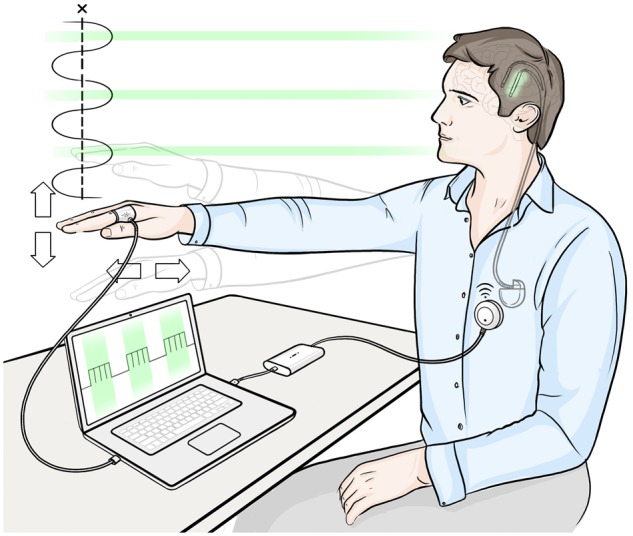
**Phase-specific stimulation.** The neurostimulator is controlled by patient’s tremor, sensed using the accelerometer attached to the tremulous hand. The green segments indicate when a burst of stimulation is applied to patient’s ventrolateral thalamus. The exact timing of stimulation onset is locked to a particular tremor phase, and the interburst frequency is equivalent to the patient’s tremor frequency.

How can neural synchrony be continuously and consistently controlled with low frequency stimulation to provide a more efficient alternative to continuous high frequency stimulation protocols? In a previous study, we have shown that continuous stimulation at patients’ tremor frequency (3–8 Hz) entrains tremor-related neural oscillations—revealing stimulation induced brief enhancement and suppression of neural synchrony reflected peripherally as transient tremor amplitude modulation (Cagnan *et al.*, 2013). In this study, we develop this critical observation into a novel stimulation strategy that enhances the efficacy of DBS by tailoring the stimulation timing to a certain phase of tremor-related neural oscillations to selectively ‘decouple’ the tremor network (Fig. 1). We demonstrate that by stimulating at overall lower frequencies but timed to interact with pathological activity, we can, in selected cases, substantially reduce the total stimulation energy delivered to essential tremor patients without compromising therapy efficacy. Such a stimulation strategy may elicit fewer side effects due to the lower energy delivered (Pedrosa *et al.*, 2014), and the fact that other rhythmic activities that are not phase-locked to the stimulation should in principle be relatively spared. Critically, we implement phase interference of tremor with DBS in a clinically tractable paradigm that derives stimulation timing from peripheral inertial sensors attached to patients’ tremulous limbs rather than sensing oscillations from the brain directly, using existing neurostimulator technology (Fig. 1) (Cagnan *et al.*, 2015*a*).

## Materials and methods

### Subjects

Essential and dystonic tremor patients with DBS implants for standard clinical indications were recruited for this study (Table 1 and Supplementary Table 1). Patients were selected using the following criterion so that objective and reliable judgements could be made about the efficacy of stimulation. Standard high frequency stimulation had to suppress tremor by >80% with charge densities <30 μQ/cm^2^/phase. This criterion was used to ensure that patient symptoms were effectively managed with standard high frequency stimulation protocols, and led to the exclusion of one patient with dystonic tremor (Supplementary Table 1 andSupplementary Fig. 1, Subject 10). Following application of the above exclusion criterion, nine patients were included in the study. Two recordings were obtained from one patient during different visits. This subject is represented as Subject 4R and 4L, denoting patient’s right and left hand, respectively. Six patients were chronically implanted (i.e. 6 months since electrode implantation), and three patients were assessed 3–6 days following electrode implantation.

**Table 1:** Neurostimulator settings

Subject	Diagnosis	Stimulation contact	Stimulation setting: high-frequency (TEED)	Stimulation setting: phase-specific (TEED)	Stimulation electrode
1	Essential tremor	0− 1+	200 µs, 1 V, 130 Hz (26 µJ)	200 µs, 1.1 V (8.7 µJ)	Left
2	Essential tremor	1− B+	90 µs, 2.3 V, 130 Hz (62 µJ)	210 µs, 2.3 V (20 µJ)	Right
3	Essential tremor	0−1+2+3+	60 µs, 2.1 V, 185 Hz (49 µJ)	180 µs, 2.1 V (25 µJ)	Left
4 R	Essential tremor	3− B+	60 µs, 1.6 V, 130 Hz (20 µJ)	210 µs, 1.4 V (9.4 µJ)	Left
4 L	Essential tremor	1− B+	60 µs, 2.7 V, 130 Hz (57 µJ)	210 µs, 2.2 V (20 µJ)	Right
5	Essential tremor	0− B+	60 µs, 2.8 V, 180 Hz (85 µJ)	210 µs, 2.6 V (36 µJ)	Left
6	Essential tremor	0− B+	60 µs, 2.6 V, 180 Hz (73 µJ)	210 µs, 2.1 V (23 µJ)	Left
7	Dystonic tremor	1− 2+	200 µs, 1.1 V, 130 Hz (31.5 µJ)	200 µs, 2 V (14 µJ)	Left
8	Dystonic tremor	1− 2+	200 µs, 3.5 V, 130 Hz (310 µJ)	200 µs, 3.5 V (44 µJ)	Left
9	Dystonic tremor	0− B+	60 µs, 3.2 V, 130 Hz (80 µJ)	210 µs, 3.2 V (39 µJ)	Left

Implanted macro-electrodes (Medtronic 3387 or 3389) have four platinum-iridium contacts, which are numbered 0, 1, 2 and 3, with 0 being the most caudal and 3 being the most rostral contact. B refers to the implanted neurostimulator case for monopolar stimulation. Total electrical energy delivered per unit time (TEED) is calculated assuming an impedance of 1000 Ω using the following formula (Koss *et al.*, 2005): TEED = [voltage^2 ^× (pulse width) × (stimulation frequency)] / impedance.

All patients gave their informed consent to take part in the study, which was approved by the local ethics committee in accordance with the Declaration of Helsinki. Patients were implanted with DBS electrodes in the ventrolateral thalamus for treatment of essential or dystonic tremor (Table 1 and Supplementary Table 1), using previously described surgical techniques (Holl *et al.*, 2010). Electrode location was confirmed by the effect of intraoperative high frequency stimulation and with postoperative imaging (CT or MRI).

### Study procedure

On the day of the recording, standard high frequency DBS was switched off and a triaxial accelerometer (Biometrics Ltd, ACL300) was attached to the metacarpophalangeal joint of the index finger of the patient’s most tremulous hand. The accelerometer signal was amplified using a Biometrics K800 amplifier and the signal from the dominant tremor axis was filtered online using a 1902 isolated pre-amplifier, which was then recorded and processed using a 1401 amplifier and Spike2 software (Cambridge Electronic Design) (recording sampling rate: 10.417 kHz). For the online filter, a 2-pole digital Butterworth filter was used with a cut-off frequency of ±2 Hz around the patient’s tremor frequency (i.e. a decrease of −12 dB per octave outside of the pass-band). The mean tremor frequency was 4.22 ± 0.25 [mean ± standard error of the mean (SEM)]. The digital 1902 filter induced a latency shift of 0.35 ms due to buffering during filtering. This latency was the same for all frequencies (1902 Cambridge Electronic Design). In addition, cross-spectral density between the filtered and unfiltered accelerometer recordings revealed that online filtering induced a 0.21 ± 0.05 (mean ± SEM) radians/Hz phase shift from f − 1 Hz to f + 1 Hz, where f denotes the average tremor frequency. The signal from the triaxial accelerometer with the highest spectral peak at the tremor frequency during the no stimulation session was defined as the dominant tremor axis. This was determined by visual inspection. This choice was corroborated *post hoc* using principal component analysis, which indicated that the axis selected as the dominant tremor axis was the principal movement axis on average 73 ± 7% of the total recording period (*n = *8; Subjects 1–6 and 8).

Patients were asked to assume a tremor provoking position, and patients’ tremor severity was recorded (i) in the absence of stimulation; (ii) during standard high frequency stimulation; (iii) during 5-s blocks of stimulation at randomly selected fixed phases; and (iv) during prolonged stimulation at a selected phase.

### Phasic stimulation

The band-pass filtered signal from the dominant tremor axis was used to determine the tremor phase in real time and to control the stimulator. To implement phase-specific stimulation, tremor phase was estimated from the tremor frequency and the timing of the preceding zero crossing. When a certain tremor phase was detected, a TTL pulse was sent to either an externalized stimulator (for the externalized patients) (Little *et al.*, 2013) or to the Nexus-D (for the chronically implanted patients) (Cagnan *et al.*, 2015*a*), which in turn delivered a burst of high frequency stimulation unilaterally for 35 ms (i.e. four to six pulses, Supplementary Fig. 2) to the ventrolateral thalamus contralateral to the most tremulous hand (Table 1). The frequency and amplitude of each pulse and the stimulation configuration (i.e. monopolar or bipolar) were based on values independently determined by the clinical team to give the best therapeutic result during continuous high frequency stimulation (Table 1). Stimulation pulse width was increased to 180–210 µs based on a previous study (Cagnan *et al.*, 2013) (Table 1). Stimulation onset was triggered by the experimenter once the patient assumed a tremor provoking position. The onset and offset of the stimulation period were defined from the TTL pulses sent to the externalized stimulator or Nexus-D.

The stimulation phase that gave rise to maximal tremor suppression was derived empirically for each patient. To this end, patients were asked to assume a tremor provoking posture for 71 s at a time. Stimulation was phase locked for 5 s to a phase value randomly chosen from 0° to 330° (resolution 30°giving 12 possible phase values; Supplementary Fig. 3A). Each phasic stimulation block was separated by 1 s of no stimulation and the order of stimulation phase was randomized between each trial ensuring that the outcome measure (i.e. change in tremor severity) was not confounded by the sequence of stimulation phase. Randomized phasic stimulation trials were repeated 6–10 times according to patient fatigue.

Following the randomized phasic stimulation trials, the most effective stimulation phase for amplitude suppression was delivered in blocks lasting on average 28.6 ± 1 s (range 17–54 s) to a subset of patients (Subjects 1, 3, 4 R, 4 L, 5, 7, and 9; Supplementary Fig. 1). As before the patients were asked to assume a tremor provoking posture prior to stimulation delivery. Stimulation at the most effective phase was repeated 1–10 times according to patient fatigue, with periods of rest lasting on average 72 ± 10 s during which stimulation was not applied. We repeated trials to check for the consistency of the effects of phase-specific DBS, and thereby to distinguish effects from spontaneous variation in tremor severity as seen in the absence of stimulation. We terminated prolonged stimulation trials on average at 28.6 ± 1 s to prevent fatigue, and in order to be able to record the effect of phase-specific DBS over multiple repetitions of the same posture.

### Data analysis

Data were analysed offline using custom-written Matlab software [MathWorks (USA)].

### Dominant tremor axis

For the *post hoc* assessment of the dominant tremor axis, recordings obtained during 5-s blocks of stimulation at randomly selected fixed phases were divided into 5-s long epochs (corresponding to stimulation periods; Supplementary Fig. 3A) and principal component analysis was applied at each epoch to determine which tremor axis had the highest coefficient. Subjects 7 and 9 were excluded from this analysis because a copy of the unfiltered accelerometer signal was not available.

### Stimulation efficacy

Triaxial accelerometer signals were down-sampled to 1000 Hz and band-pass filtered ± 2 Hz around the tremor frequency with peak amplitude using a second order Butterworth filter applied forwards and backwards. Instantaneous tremor phase and envelope were estimated using the Hilbert Transform (Cagnan *et al.*, 2013, 2014). The change in tremor severity was summarized as the average change in tremor envelope at the last second of the stimulation block (4–5 s) with respect to average tremor severity 1 s prior to the onset of each stimulation block (Supplementary Fig. 3B). These measures were normalized by the average tremor severity 1 s prior to the onset of each stimulation block (Supplementary Fig. 3B), furnishing a normalized measure between −1 and a positive number, where −1 indicates complete tremor suppression, 0 indicates no change in tremor and a positive number indicates tremor amplification. The use of the 1 s prior to onset of each stimulation block meant that there was no preceding washout period as stimulation blocks were only separated by 1 s. However, extending the duration of tremor provoking posture beyond 71 s at a time was considered too fatiguing, and the randomization of the order of stimulation phases across repeated trials of tremor provoking posture should have acted to reduce any systematic bias from failure to washout. This lack of washout could have led to an underestimation of the effect size.

Similarly for prolonged phase-specific stimulation, the change in tremor severity was summarized as the average tremor envelope at the last second of the stimulation block with respect to average tremor severity 1 s prior to the onset of each stimulation block, normalized by the average tremor severity 1 s prior to the onset of each stimulation block. Phase tracking stability could reduce during phase-specific stimulation as patients’ tremor was suppressed, because of phase instability of weak tremor or due to failure to estimate phase with weak tremor at stimulation onset (Supplementary Fig. 4).

### Time course of the stimulation effect

To derive the time point when 50% of the maximum stimulation effect for each trial was reached; we first fit a sigmoid function to the tremor envelope, which was down-sampled to 4 Hz (Supplementary Fig. 5). Instantaneous tremor severity (i.e. tremor envelope) changes at a rate slower than the tremor frequency. Therefore, down sampling the tremor envelope by the average tremor frequency would not compromise the information content (average tremor frequency was 4.22 Hz across all subjects). For each prolonged phase-specific DBS trial, the time point corresponding to the ‘50% of the maximum stimulation effect’ was derived to quantify the temporal dynamics of the stimulation effect (Supplementary Fig. 5). For instance, for 60% tremor suppression in a trial, ‘50% of the maximum stimulation effect’ would correspond to the time point when tremor severity would reduce by 30%.

### Outliers

To eliminate changes in tremor envelope due to voluntary movement and due to posture changes, episodes during which tremor envelope dropped below (mean − 1 standard deviation) for >10 s were excluded from analysis. This criterion was applied to recordings obtained during random phasic stimulation and to recordings obtained in the absence of stimulation.

Stimulation epochs were excluded from analysis if tremor severity was ≤0.2 m/s^2^ at the onset of the prolonged phase-specific stimulation. This criterion was applied to ensure that (i) tremor signal to noise ratio was high enough to accurately estimate phase; and (ii) stimulation efficacy was estimated only when patients were tremulous in the absence of stimulation.

### Statistical analysis

#### Surrogate distribution

Whether delivering stimulation at a certain tremor phase significantly modulated tremor was determined with respect to tremor variability when stimulation was not applied to the patient. To this end the tremor envelope was divided into 50 000 randomly chosen 5-s long segments and the change in tremor severity was calculated as described in the previous section. *N* of these tremor change values were randomly chosen from this distribution and the medians of these tremor changes were taken. *N* was determined according to the number of trials available from each patient. This procedure was repeated 1 000 000 times for each patient, giving rise to a surrogate distribution for changes in tremor severity when stimulation was not applied. Significance of a tremor change observed at a stimulation phase was assessed with respect to the surrogate distribution, using *z-*score, and significance was corrected for multiple comparisons using the Bonferroni method. Recordings, during which stimulation was not applied, lasted on average 158 ± 31 s, divided into segments of tremulous posture lasting on average 34 ± 3 s. A segment of tremulous posture was defined as time segments during which the filtered tremor envelope (low pass filtered with cut-off frequency of 0.1 Hz) was elevated above the average tremor severity for >10 s.

## Results

In this study, we aimed to experimentally validate whether neural oscillators, underlying pathologies such as tremor, can be moved away from critical temporal relationships that reinforce neural synchrony using phase-specific stimulation. Such interactions are of great interest in order to provide a more efficient alternative to continuous high frequency stimulation protocols. We have previously shown that continuous stimulation at patients’ tremor frequency, increased tremor regularity (Cagnan *et al.*, 2013). Transient alignments between thalamic stimulation and patients’ tremor modulated tremor severity on average by 10% (Cagnan *et al.*, 2013). Here we determine whether such modulatory effects can be harnessed consistently to induce clinically relevant symptom relief through phase-specific stimulation.

### Essential tremor

#### Short-term effect of phase-specific thalamic stimulation

In this study, we first determined whether phase locking thalamic stimulation to a particular angle in the tremor cycle would consistently modulate tremor amplitude in a group of essential tremor patients (Subjects 1–6). Every tremor cycle, a burst of high frequency pulses was delivered to the ventrolateral thalamus, phase-locked to a certain tremor phase (Fig. 1, see Table 1 for pulse amplitude, width, and frequency). Each burst lasted 35 ms, spanning ∼60° of the tremor cycle. For each trial, the stimulation phase was randomly selected from 12 possible equally spaced phase values, and stimulation at each phase lasted for 5 s before being repeated at another randomly selected phase a second later (Supplementary Fig. 3A).

**Figure 2:**
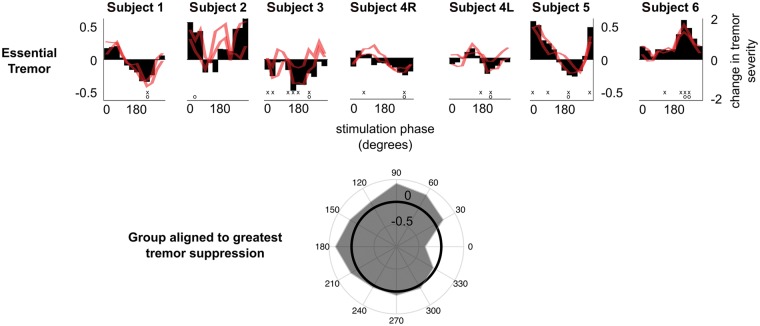
**Tremor amplitude can be consistently modulated with phase-specific thalamic stimulation in essential tremor patients.** Black bars indicate the median amplitude change at each stimulation phase at the dominant tremor axis, while the red lines show the median amplitude change at the other two tremor axes which were not phase-tracked to control stimulation. Tremor severity is normalized such that −1 indicates complete tremor suppression, 0 indicates no change in tremor severity and a positive number indicates tremor amplification. Note that, for presentation purposes, median stimulation phase-amplitude relationships have been smoothed using a moving average filter with a span of three stimulation phases. However, all ranges presented in the main text and statistical analyses, involved data prior to smoothing. ‘x’ indicates stimulation phases that gave rise to a significant modulation in tremor severity with respect to the no stimulation condition while ‘o’ indicates the stimulation phase that gave rise to the most consistent change in tremor amplitude across all trials. Significance was tested with respect to the surrogate distribution derived from the no stimulation condition, and corrected for 12 effective comparisons using Bonferroni correction. *Bottom*: The average phase-amplitude relationship across essential tremor patients (and both sides in the case of Subject 4) realigned such that 0° would correspond to the stimulation phase that gave rise to the greatest tremor suppression. At the group level, there was a main effect of stimulation phase on tremor severity (Friedman’s test *P = *0.0026).

Considering the first case presented in Fig. 2, stimulation significantly reduced tremor severity (*P < *0.0001) at the end of phase-specific stimulation delivered in 5-s blocks at a phase of 240° with respect to tremor in the principal movement axis. Significance was tested with respect to the surrogate distribution derived from the no stimulation condition, and corrected for 12 effective comparisons using Bonferroni correction. With stimulation delivered at 240° in this patient, tremor amplitude was reduced in seven of nine trials at the end of each stimulation block, with respect to tremor severity at the onset of each block.

On average 35.75% (range 8–51%) tremor-suppression was observed in essential tremor patients at the end of phase-specific stimulation blocks (Fig. 2), increasing to 38.5% (range 28–51%) if only those five subjects displaying significant suppression were considered. Note that we excluded tremor amplification from this reported range as amplifying phases were not relevant for a positive therapeutic effect, and are not hereafter considered as potential stimulation phases for sustained phase-specific stimulation. Realigning median stimulation phase-amplitude relationships derived from each case such that 0° would correspond to the stimulation phase that gave rise to the greatest tremor suppression, we tested whether different stimulation phases induced the same effect on tremor severity. Across all essential tremor patients, there was a main effect of stimulation phase on tremor severity (Friedman’s test *P = *0.0026, *df* = 11).

Phase-specific stimulation tended to induce similar phase dependent amplitude changes in the two non-dominant tremor axes (red traces in Fig. 2) compared to the dominant axis. However, this was not exclusively the case, and the non-dominant tremor axes, such as in Subject 2 could have independent phase-amplitude profiles, alluding to the existence of several independent neural oscillators controlling different directions of limb movement during a tremor episode.

Finally phase-specific DBS in Subject 6 was unusual in that it significantly, and exclusively, amplified the patient’s tremor with respect to instantaneous changes observed in tremor severity in the absence of stimulation. This was in contrast to other phase-amplitude profiles, where certain stimulation phases increased tremor severity but most often stimulation phases reduced tremor severity. Another intriguing aspect of this patient’s response to phase-specific stimulation was that at the end of phase-specific stimulation, regardless of stimulation phase, the patient’s tremor dissipated (Supplementary Fig. 6). While there were not any obvious clinical differences in this patient (Supplementary Table 1), the above response to phase-specific DBS is potentially suggestive of direct activation of the cortico-spinal tract by stimulation. Thus when stimulation was applied at an appropriate angle with respect to the ongoing tremor activity, stimulation may have enhanced ongoing tremor activity through an interaction at the level of the spinal cord.

### Effects of longer phase-specific stimulation

In this study, our ultimate goal was to determine whether phase-specific stimulation could induce clinically significant tremor suppression more efficiently than conventional high frequency thalamic stimulation. Clinically significant tremor relief in the hand was operationally defined as residual tremor with amplitude of ≤0.2 m/s^2^, which corresponds to symptom severity of ≤1 on the Bain and Findley tremor severity scale (Bain *et al.*, 1993). For comparison, a normative study of healthy subjects aged 15 to 80 years old reported tremor amplitudes of up to 0.35 m/s^2^, and that hand tremor of 0.07 m/s^2^ was just about visible (Wade *et al.*, 1982). Only Subjects 1, 3, 4R, 4L and 5 exhibited significant tremor suppression during 5-s long stimulation trials (Fig. 2). Significance was tested with respect to the surrogate distribution derived from tremor recordings obtained when stimulation was not applied to patients, and corrected for 12 effective comparisons using Bonferroni correction. We therefore investigated the suppressive effects of phase-specific DBS in Subjects 1, 3, 4R, 4L, and 5 at or close to the optimal suppressive phase determined from the 5-s phase locked trials (Fig. 2 and Supplementary Fig. 1). For all cases, the test of more prolonged phase-specific stimulation included the phase reducing tremor most significantly during randomized 5-s long stimulation trials, given that stimulation covered 60° of the tremor cycle.

**Figure 3:**
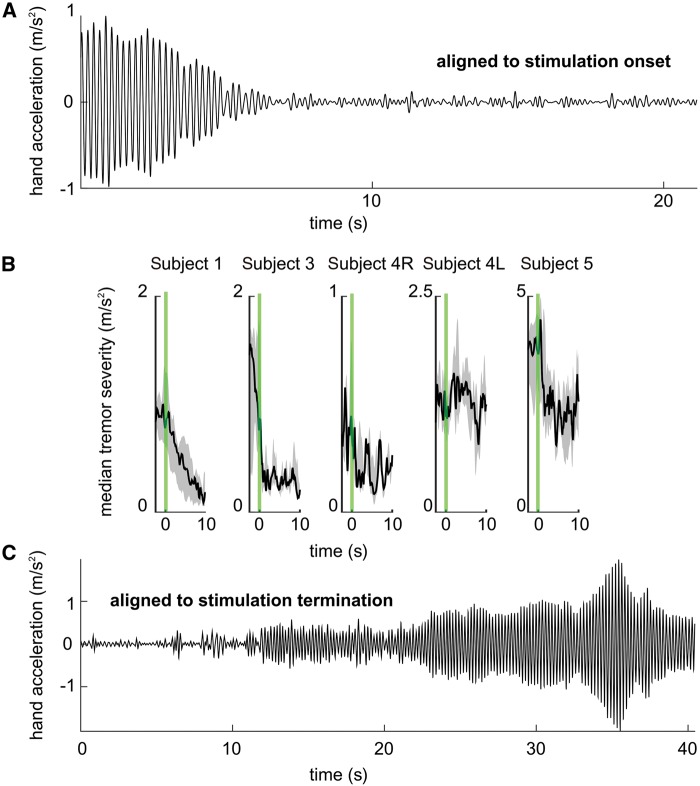
**Phase-specific stimulation can induce clinically significant tremor suppression more efficiently than conventional high frequency thalamic stimulation.** (**A**) Subject 1’s hand acceleration (m/s^2^) during a single trial of phase-specific stimulation aligned to stimulation onset. (**B**) Black lines indicate the median tremor intensity observed, while shaded regions indicate the 25th–75th percentiles across different trials. Repeated trials, including those during which the patient’s principal movement axis was different from the tremor axis tracked for stimulation, depicted in **B** were aligned to stimulation onset. Green lines indicate onset of phasic stimulation. Stimulation at the optimal phase for tremor suppression lasted on average 28.6 ± 1 s. Subject 1: across seven trials, median tremor intensity at the end of prolonged phase-specific stimulation at 240° was 0.08 m/s^2^ (IQR 0.06–0.08–0.21 m/s^2^). Subject 3: tremor intensity reduced to 0.21 m/s^2^ (IQR 0.15–0.21–0.28 m/s^2^) during phase-specific stimulation at 150° across nine trials. Subject 4R: tremor intensity reduced to 0.19 m/s^2^ during phase-specific stimulation at 270° (IQR 0.17–0.19–0.33 m/s^2^). Subject 4 L: tremor intensity reduced to 1.35 m/s^2^ during phase-specific stimulation at 240° (IQR 0.86–1.35–1.67 m/s^2^). Subject 5: tremor intensity reduced to 2.02 m/s^2^ during phase-specific stimulation at 210° (IQR 0.87–2.02–2.56 m/s^2^). For all subjects, high frequency stimulation resulted in tremor suppression ≤0.23 m/s^2^ (Subject 1: 0.12 m/s^2^, Subject 3: 0.09 m/s^2^, Subject 4R: 0.23 m/s^2^, Subject 4L: 0.09 m/s^2^ Subject 5: 0.03 m/s^2^). (**C**) Subject 1’s hand acceleration (m/s^2^) during a single trial of phase-specific stimulation indicating the delayed return to the prior tremor amplitude following stimulation termination.

On average tremor was suppressed by 64% when a patient’s principal movement axis was the same as the tremor axis tracked to control the timing of phase-specific DBS. If all trials were taken into account, including those during which the patient’s principal movement axis was different from the tremor axis tracked for stimulation, on average tremor was suppressed by 56% of its prestimulation amplitude, as measured after on average 28.6 ± 1 s of phase-specific DBS (Fig. 3). This difference highlights the importance of tracking changes in the principal movement axis to maximize the effect of phase-specific DBS. In Subject 5, as phase-specific DBS suppressed the patient’s tremor at 4.25 Hz, a different oscillation emerged at 2.8 Hz (Supplementary Fig. 7). Intriguingly, the slower tremor oscillation was not present when stimulation was not applied or during 5-s long phase-specific stimulation trials (Fig. 2 and Supplementary Fig. 7), and only emerged once the main tremor oscillation at 4.25 Hz was suppressed (Supplementary Fig. 7). It should be noted that values reported for Subject 5 correspond to the temporal dynamics of the main tremor oscillation between 3.5 and 6.5 Hz (Table 2).

**Table 2:** Effect of prolonged phase-specific DBS in essential tremor patients

	Principal movement axis tracked	All trials					
Subject	Tremor suppression	Tremor suppression	Tremor severity phase-specific DBS	50% of the maximum stimulation effect	Average tremor severity high frequency DBS	Average tremor severity in the absence of DBS	Frequency band
1	90.06% (*n = *4)	86.94% (*n = *7)	IQR 0.06–0.08–0.21 m/s^2^	4.25 s (R = 0.93)	0.12 m/s^2^	1.9 m/s^2^	3–7 Hz
3	78.79% (*n = *8)	76.85% (*n = *9)	IQR 0.15–0.21–0.28 m/s^2^	0.62 s (R = 0.69)	0.08 m/s^2^	4.4 m/s^2^	3–7 Hz
4R	57.68% (*n = *1)	57.68% (*n = *3)	IQR 0.17–0.19–0.33 m/s^2^	3.75 s (R = 0.65)	0.23 m/s^2^	1.3 m/s^2^	2.5–6.5 Hz
4L	26.56% (*n = *2)	6% (*n = *4)	IQR 0.86–1.35–1.67 m/s^2^	17.5 s (R = 0.14)	0.09 m/s^2^	2.66 m/s^2^	2.5–6.5 Hz
5	65.30% (*n = *4)	52.38% (*n = *9)	IQR 0.87–2.02–2.56 m/s^2^	2.12 s (R = 0.46)	0.03 m/s^2^	5.3 m/s^2^	3.5–6.5 Hz

Median tremor suppression is indicated across all, and a subset of the trials during which the principal movement axis was the same as the tremor axis being tracked together with the interquartile range (IQR) for tremor severity at the end of phase-specific DBS trials. Median time point when ‘50% of the maximum stimulation effect’ is reached indicates the temporal dynamics of the tremor suppression. The number of trials is indicated by *n*, while R indicates the median Pearson’s correlation coefficient between the tremor envelope and the sigmoid function fitted.

We derived the time point corresponding to ‘50% of the maximum stimulation effect’ for each trial to quantify further the time course of tremor modulation during phase-specific DBS (Supplementary Fig. 5). The median time point corresponding to the ‘50% of the maximum stimulation effect’ was 3.75 s for Subjects 1, 3, 4R, 4L, and 5 (Table 2).

In Subjects 1, 3, and 4R, clinically significant tremor relief was attained, with median tremor severity of ≤0.2 m/s^2^ at the end of phase-specific stimulation (Table 2). For Subjects 1 and 3, this corresponded to a drop in tremor severity from a score of 4 or 5 to 1 or less on the Bain and Findley tremor rating scale (Bain *et al.*, 1993). For Subject 4R, tremor severity reduced from 2 to ≤1. For Subjects 1 and 4R, tremor suppression achieved during phase-specific DBS was not different from that observed during conventional high frequency DBS, while for Subjects 3, 4L and 5 these two levels were significantly different (one sample student’s *t*-test Subject 1: *n = *7, *P = *0.7381, Subject 3: *n = *9, *P = *0.0041, Subject 4R: *n = *3, *P = *0.7028, Subject 4L: *n = *4, *P = *0.0167, Subject 5: *n = *9, *P = *0.01). Note that there was a delayed return to the prior tremor amplitude following offset of phase-specific stimulation, suggestive of delayed washout following effective stimulation (Fig. 3C). For Subjects 4L and 5, the degree of suppression of the target tremor (27 and 65%, respectively) was confounded by changes in the principal movement axis, and by emergence of independent tremor oscillators following the suppression of the main tremor oscillator (Table 2 and Supplementary Fig. 7).

Achieving tremor suppression while delivering lower energy is particularly relevant to minimize high frequency induced side effects (Pedrosa *et al.*, 2014). For comparison, continuous thalamic stimulation at 130 Hz resulted in clinically significant tremor relief in all of the above cases, but involved a total electrical energy delivered per unit time (TEED) of 47 µJ (range 20–85 µJ), compared to 20 µJ (range 8.7–26 µJ) during phase-specific stimulation (42% of the TEED during high frequency stimulation, range 33–51%) (Table 1). Thus the efficiency of tremor suppression, defined as the per cent tremor suppression divided by the TEED, was 1.7 times greater during phase-specific stimulation when patient’s principal movement axis was the same as the tremor axis tracked to control the timing of phase-specific DBS (during phasic stimulation 4.6% change in tremor severity per µJ; during high frequency stimulation 2.5% change in tremor severity per µJ), and 1.4 times greater when all trials were taken into account including those during which patient’s principal movement axis was different from the tremor axis tracked for stimulation.

### Dystonic tremor

Might tremor involving a different pathophysiological circuit respond differently to phase-specific stimulation? To answer this we explored whether phase-specific stimulation of the thalamus would be effective in patients with dystonic tremor. Unlike essential tremor, the tremor circuit in this condition is thought to involve the basal ganglia output nucleus, the internal segment of the globus pallidus—as evident from the beneficial long term effects of pallidal DBS, and the coherence between dystonic muscle activity and pallidal local field potentials (McAuley and Rothwell, 2004; Sharott *et al.*, 2008; Elble, 2013; Hedera *et al.*, 2013).

**Figure 4:**
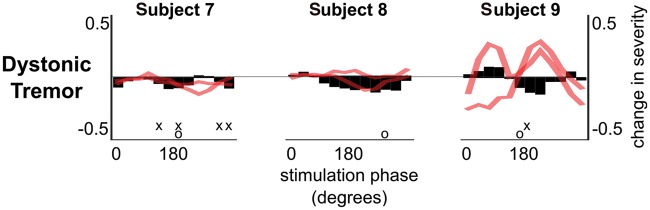
**Tremor amplitude can be consistently modulated with phase-specific thalamic stimulation in patients with dystonic tremor.** Black bars indicate the median amplitude change at each stimulation phase at the dominant tremor axis, while the red lines show the median amplitude change at the other two tremor axes, which were not phase-tracked to control stimulation. ‘x’ indicates stimulation phases that gave rise to a significant modulation in tremor severity with respect to the no stimulation condition while ‘o’ indicates the stimulation phase that gave rise to the most consistent change in tremor amplitude across all trials. Significance was tested with respect to the surrogate distribution derived from the no stimulation condition, and corrected for 12 effective comparisons using Bonferroni correction. Note that, for presentation purposes, median stimulation phase-amplitude relationships have been smoothed using a moving average filter with a span of three stimulation phases. However, all ranges presented in the main text, and statistical analyses involved data prior to smoothing.

Similar to the essential tremor patients, we locked the stimulation phase to a randomly selected value from 12 possible equally spaced phase values, and applied stimulation at each phase for 5 s in three patients with dystonic tremor (Supplementary Fig. 3A). Phase-specific thalamic stimulation was able to modulate tremor severity in Subjects 7 and 9. Although significant, tremor-suppression was on average only 20% (range 14–26%), compared to an average of 35.75% suppression in essential tremor (Figs 2 and 4).

In Subjects 7 and 9, tremor suppression reached 43% (*n = *1) and 16% (*n = *5) when phase-specific stimulation was applied for longer periods of time. The median time point corresponding to the ‘50% of the maximum stimulation effect’ was 1.75 s, and 13.9 s (in Subjects 7 and 9, respectively). Tremor suppression was again less than in essential tremor where on average tremor was suppressed by 64% of its pre-stimulation amplitude. It also did not compare favourably with the 85% and 96% tremor suppression achieved with high frequency stimulation in these two respective dystonic patients. Although these results need confirmation in more patients with dystonic tremor, they are consistent with the hypothesis that phase-specific stimulation efficacy depends on the nature of the underlying tremor network. The latter assumes that the efficiency of phase tracking was similar in patients with essential and dystonic tremor, so that the difference does not arise at the level of delivery of the intervention but through differing tremor network susceptibility. Using the instantaneous tremor phase derived from the band-pass filtered tremor signal, we determined the phase tracking efficacy of our algorithm. We computed the vector length of the average tremor phase at which stimulation was delivered during 5-s long stimulation blocks across all trials. If stimulation was delivered on average at the same tremor phase across all trials, then vector length would be 1, otherwise, if there was not phase consistency between trials, then vector length would be 0. Individual phase tracking efficiency data are given in Supplementary Table 2. Two of the three cases with the lowest tracking efficiency were also the only two patients not to show significant phase-amplitude effects. Thus phase tracking efficiency may well prove an important determinant of efficacy, but the fact that these patients were drawn from both patient groups suggests that this may not completely explain any disease-specific differences in responses to phase-specific stimulation. Underscoring this was the fact that Subject 7, with dystonic tremor, had the highest phase tracking efficiency of all, and yet tremor suppression still only reached 43% with longer stimulation periods. Thus to realize clinically significant tremor suppression in patients with dystonic tremor the amount of energy delivered during phase-specific stimulation may need to be increased by either increasing the number of pulses delivered per tremor cycle or by increasing the stimulation amplitude.

### Effect of phase-specific deep brain stimulation may depend on the underlying neural network

It has been shown previously that tremor amplitude strongly depends on how stable tremor frequency is, reflecting the resonant properties of the underlying tremor oscillators (Cagnan *et al.*, 2014; Brittain *et al.*, 2015). Accordingly, we explored whether phase-specific stimulation can act to shift tremor frequency and thereby modulate tremor amplitude.

**Figure 5:**
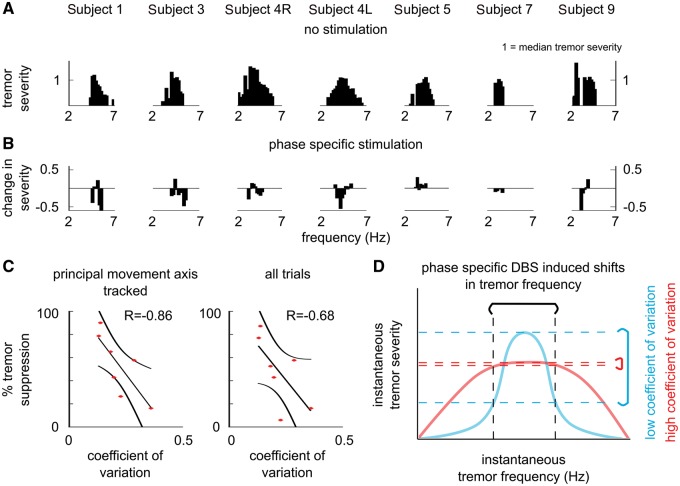
**Tremor’s resonant properties may determine how well a patient will respond to phase-specific DBS.** (**A**) Patients’ tremor severity varied with the instantaneous tremor frequency. (**B**) At the end of 5-s phase-specific stimulation blocks (Figs 2 and 4), tremor frequency either remained at the median tremor frequency or changed (Supplementary Fig. 8). When patients’ tremor frequency either increased or decreased, a reduction in tremor severity tended to be observed. It should be noted that changes in tremor severity were arranged according to the stimulation phase in Figs 2 and 4, while in **B** these changes were arranged according to the instantaneous tremor frequency. (**C**) Fitting a Gaussian to the instantaneous tremor frequency and severity relationship (shown in **A**), revealed that those subjects who benefited the most from prolonged phase-specific DBS (trialed on Subjects 1, 3, 4R, 4L, 5, 7 and 9), also had the smallest coefficient of variation of tremor amplitude over different tremor frequencies. Coefficient of variation and tremor suppression were significantly correlated when the principal movement axis was the same as the tremor axis tracked to control DBS (*P = *0.0238). It should be noted that, for Subjects 7 and 9, the principal movement axis could not be derived. Therefore, the median tremor suppression observed across all trials was used when estimating the relationship between the coefficient of variation and the per cent tremor suppression during phase-specific DBS. R indicates the Spearman’s rank correlation coefficient between the coefficient of variation and per cent tremor suppression. (**D**) Resonant properties of oscillators underlying tremor. For tremors with high coefficient of variation, changes in tremor frequency induced by phase-specific DBS would lead to a relatively small change in tremor severity. However, for tremors with low coefficient of variation, small changes in tremor frequency would lead to a relatively big change in tremor severity.

As highlighted in Fig. 5A, in essential and dystonic tremor patients, tremor amplitude varied with instantaneous tremor frequency. Fitting a Gaussian distribution to the relationship between instantaneous tremor frequency and amplitude (normalized to the individual median tremor amplitude) (Fig. 5A), we estimated the coefficient of variation for each subject. For some patients, the coefficient of variation was relatively small (e.g. Subjects 1, 3 and 5) while for others, tremor amplitude remained elevated over a broad range of frequencies.

Several theoretical and electrophysiological studies have highlighted that stimulation timing together with membrane properties of a neuron determine whether a stimulus is going to delay the spiking activity of a rhythmically firing neuron (i.e. reduce the firing frequency), or induce spiking activity earlier (i.e. increase the firing frequency) (Ermentrout, 1996; Smeal *et al.*, 2010; Wilson and Moehlis, 2014). Dividing the 5-s long phase locked stimulation trials according to tremor frequency at the end of each stimulation block, regardless of stimulation phase, revealed that phase-specific DBS may act on this fundamental neural property—depending on the timing of stimulation with respect to the ongoing tremor oscillation, phase-specific DBS may either increase or reduce the frequency of the tremor oscillation (Fig. 5B). How much a patient’s tremor varied with instantaneous changes in tremor frequency (Fig. 5A), may also determine how easily a patient’s tremor could be suppressed with phase-specific DBS. Patients’ who benefitted the most from prolonged phase-specific stimulation were also those with the smallest coefficient of variation (Fig. 5C and D).

## Discussion

Here, we explore phase-specific thalamic stimulation in a group of tremor patients. In selected patients with essential tremor, as opposed to those with dystonic tremor, prolonged phase-specific stimulation could achieve clinically beneficial tremor suppression as could state-of the art high frequency DBS, yet with on average 42% of the TEED necessary with the latter.

### Deep brain stimulation

DBS has proven a successful treatment for several neurological disorders (Benabid *et al.*, 1991; Rodriguez-Oroz *et al.*, 2005; Okun, 2012; Miocinovic *et al.*, 2013). Current stimulation protocols, which involve continuous stimulation at high frequencies (130–180 Hz), are thought to mimic the effect of lesioning by modulating neural activity patterns, and creating a reversible informational lesion (Hashimoto *et al.*, 2003; McIntyre *et al.*, 2004; Agnesi *et al.*, 2013). An alternative approach to increase the efficacy of DBS is to apply high frequency stimulation when a disease biomarker exceeds a preset threshold (Brice and Mclellan, 1980; Graupe *et al.*, 2010; Basu *et al.*, 2013; Little *et al.*, 2013; Yamamoto *et al.*, 2013; Herron and Chizeck, 2014). While such a stimulation strategy could result in up to 50% less stimulation (Graupe *et al.*, 2010), it does not exploit the fundamental neural properties that give rise to sustained synchrony driving disease symptoms and may therefore not maximize selectivity (Fries, 2005; Cagnan *et al.*, 2015*b*). In the case of on-demand, thresholded tremor stimulation this would not be clinically useful where stimulation, whether phase-specific or continuous high frequency stimulation, has a long time-constant delaying its effect.

### Phase-specific stimulation

Conceptually, the neural circuits underlying pathological tremors can be reduced to networks of coupled oscillators on two scales; a local cortical or subcortical level where coupled oscillators may be neurons or microcircuits (Hua and Lenz, 2005), and a systems level whereby tremor is underpinned by large-scale networks of distributed oscillators (Schnitzler *et al.*, 2009). Oscillations on both scales may be sustained or dissipated by the same two fundamental processes. The first is passive, rapid in its effect and rooted in the fact that there are phases at which stimulation can decrease or increase the period (i.e*.* instantaneous frequency) of an oscillator (Ermentrout, 1996; Smeal *et al.*, 2010). In systems of coupled oscillators this can effectively modulate how close the system is to its peak resonance and maximum amplitude (Cagnan *et al.*, 2014). Figure 5 illustrates this very effect; those phases of stimulation that pull the instantaneous frequency of the tremor away from that giving the peak tremor amplitude are associated with diminished tremor amplitude.

The second process whereby oscillatory activity may be sustained or dissipated is spike-timing dependent plasticity, which may operate over longer time scales to modulate tremor. By systematically repeating electrical impulses at a given phase we are disturbing the temporal relationship between physiological synaptic inputs sustaining the tremor and output in the form of discharges. This creates the potential for spike-timing dependent potentiation or depression, and strengthening or weakening of synchrony.

It is likely that phase-specific thalamic stimulation acted through both the above mechanisms, albeit to different degrees in different patients. In some patients initial tremor suppression (and recovery on cessation of stimulation) was rapid (Fig. 3B), which probably relates to the first passive process described above; whereas in others a slower delayed suppression (and recovery) suggestive of spike-timing dependent plasticity was evident (Fig. 3B).

Tremor is posited to be the composite output of a network of local and distributed neural oscillators (Hua and Lenz, 2005; Schnitzler *et al.*, 2009). Oscillators can be divided by their response to stimulation, in the form of a phase response curve. In the current study tremor oscillators exhibited a type II phase response curve in response to ventrolateral thalamus stimulation in which the instantaneous tremor frequency could either increase or decrease depending on the precise timing of stimulation (Supplementary Fig. 8). The significance of this is that neurons with a type II phase response curve may shift into a synchronized state, thereby promoting tremor, whereas those that display a PRC type I phase response curve cannot (Ermentrout, 1996).

Phase-specific stimulation pioneered here should be distinguished from coordinated reset—a stimulation technique that aims to desynchronise locally coupled oscillators through spatiotemporally patterned stimulation motifs that are delivered open-loop, without the need for phase tracking (Tass *et al.*, 2012; Adamchic *et al.*, 2014). In the absence of closed-loop protocols, the effects are likely to be relatively unselective, as any locally synchronized oscillatory activity, physiological or pathological, is likely to be disrupted (see below).

### Variable tremor responses

Excessive synchrony of the brainstem-cerebello-thalamo-cortical loop is implicated in essential tremor pathophysiology (Llinás and Volkind, 1973; Schnitzler *et al.*, 2009). In recent years, Purkinjee cell loss (Louis *et al.*, 2011) and brainstem Lewy body disease (Louis, 2009) have been reported in a subset of essential tremor patients, further supporting the hypothesis that brainstem-cerebellar dysfunction plays a crucial role in essential tremor pathophysiology. Dystonic tremor pathophysiology is a lot less understood (Elble, 2013). Regions implicated in dystonic tremor pathophysiology are generally based on the functional neurosurgical targets, and include the ventrolateral thalamus and the internal segment of the globus pallidus. While both essential and dystonic tremor can be effectively treated with ventrolateral high frequency stimulation, only selected patients with essential tremor showed marked improvement with sustained phase-specific stimulation. Despite our small sample it is tempting to conclude that differences in underlying pathophysiology might underlie some of this difference in responsiveness (Deuschl and Bergman, 2002).

Within a given pathophysiology, how finely tuned a patient’s tremor is to a certain frequency band may be linked with the efficacy of phase-specific stimulation. Patients who benefited the most from this stimulation protocol also displayed narrow resonant characteristics as assessed from the relationship between instantaneous tremor frequency and amplitude when patients were not receiving stimulation (Fig. 5A and C). Cellular characteristics may help explain both this relationship and stimulation effects. Thalamocortical neurons exhibit rhythmic bursting activity owing to their membrane channel conductances (Cagnan *et al.*, 2009). The frequency of rhythmic neural activity depends on the amount of excitatory (e.g. from the motor cortex and cerebellum) and inhibitory input (e.g. from the internal segment of globus pallidus) a thalamocortical neuron receives. It is also these membrane dynamics that determine how much the frequency of rhythmic neural activity would increase or decrease when a neuron receives a stimulus, potentially determining the response of a patient to phase-specific DBS.

Other factors that might influence the response to phase-specific stimulation may directly manifest in the tremor itself. In particular, tremor irregularity might compromise phase-locking, as might the existence of multiple tremor oscillators within a given limb (Pedrosa *et al.*, 2012). To achieve complete tremor suppression during conventional high frequency DBS, stimulation contacts and parameters (e.g. pulse width and amplitude) are selected to modulate all independent tremor oscillators within the ventrolateral thalamus (Pedrosa *et al.*, 2012). During phase-locked DBS, a given stimulation phase may have a different modulatory effect on different independent tremor oscillators (Table 2 and Supplementary Fig. 7). For instance, Subjects 2 and 9 in particular had independent phase-amplitude profiles in dominant and non-dominant tremor axes, so that the optimal stimulation phase for tremor suppression in the dominant tremor axis would not suppress tremor in the remaining axes (Figs 2 and 4). In such cases the degree of tremor suppression achievable by the present technique may be relatively limited, as residual tremor in non-dominant tremor axes may persist. However, in the future this could be addressed by tracking the principal movement axis over time, and adjusting the stimulation phase at each tremor cycle according to the movement axis with the strongest tremor severity in the previous tremor cycle. Similarly, if thalamic recordings were used to determine the most effective stimulation timing, the strength of different tremor oscillators in the ventrolateral thalamus could be used to determine the stimulation phase in real-time. As highlighted in Supplementary Fig. 7, when the dominant tremor is suppressed with phase-specific DBS, an independent tremor oscillation may emerge in some subjects (here one of seven cases), further highlighting the importance of tracking independent tremor oscillators. Still this very dependence on the precise phase relationship of stimulation with the target oscillation raises the possibility of heightened selectivity and an improved side-effect profile with such stimulation; physiological activities, even of similar frequency, will tend to be spared in so far as they are unlikely to be phase locked to the tremor oscillations. The lower energy delivery during phase-specific stimulation will potentially further reduce the likelihood and severity of side effects. It is this potential for fewer side effects that should motivate further exploration of phase-specific stimulation in the future.

### Translation into clinical application

In this study we have demonstrated the potential of phase-specific thalamic stimulation in some patients with essential tremor. Above, we have discussed why not all patients may experience the same level of effect, and the success of any translation of phase-specific thalamic stimulation in to therapy is likely to rest on careful patient selection. Within our small cohort there was the suggestion that those patients with poorly tuned tremor or multiple tremor oscillators within a given limb might not be so amenable to this treatment approach in its current form. Even in those patients with a single dominant tremor oscillator in one limb the lack of tremor coherence between limbs means that more than one independent control system may be necessary where tremor is symptomatic in more than one limb (Raethjen *et al.*, 2000; Ben-Pazi *et al.*, 2001). Another issue requiring discussion is the time course of the response to phase-specific stimulation. It took a median of 3.75 s for 50% of the maximum stimulation effect to be realized in patients with essential tremor. On the face of it, this time may be too short if stimulation were to start as tremor established itself on assumption of a tremor provoking position; tremor amelioration would be delayed. However, our experimental estimates of tremor responsiveness do not capture how stimulation might be delivered in practice. The intention is not to ever discontinue stimulation; i.e. not to use it as an on-demand system, at least during waking hours. This means that stimulation will proceed even when tremor is clinically insignificant. We found that once tremor was suppressed to minimal levels (≤0.2 m/s^2^ or equivalent to ≤1 of 10 on the Bain and Findley tremor rating scale; Bain *et al.*, 1993) phase-specific stimulation was effective in maintaining suppression. This is interesting as phase estimation will have been degraded under these circumstances, suggesting that stimulation may not need to be consistently delivered at the optimal phase every tremor cycle to hold suppressed tremor networks in check. The system proposed therefore would be on all the time, holding weak tremor in check through sparse stimulation at the optimal phase, and able to overcome breakthrough tremor through more regular phase-specific simulation when tremor provoking postures were assumed. This makes us hopeful that in appropriately selected patients the current stimulation approach will serve to suppress both established tremor and prevent weak or absent tremor from establishing itself. Our results suggest that such control could potentially still be achieved with less than half the energy expended in conventional DBS in some patients. The next step is to demonstrate that even in responding patients phase-specific stimulation remains effective and reproducible over time and across the diverse rest, postural and action requirements of everyday life. This will require prolonged trials in active patients. These trials can also serve to test whether the potential for diminished side-effects is realized or not.

## Conclusion

We have demonstrated that phase-specific stimulation with bursts of pulses repeated at tremor frequency can achieve clinically significant tremor suppression in some tremulous patients despite the delivery of substantially less energy than conventional high frequency stimulation. Moreover, such control could be achieved in patients with existing chronically implanted devices through peripheral tremor tracking and telemetry. However, our study cohort was relatively small and heterogeneous, and so replication of the core findings in further cohorts is a priority. Also critical will be to establish that tremor suppression is sustained over time and during activities of daily living, and to determine whether phase-specific stimulation is associated with less speech and balance impairment than conventional high frequency stimulation. At the same time, given the variability in responses, it will be important to better determine how to select those patients that are most likely to respond with the present control algorithm, and those patients that might need dynamic phase-specific stimulation that tracks changes in oscillators. Nevertheless, the demonstration that electrical stimulation that is temporally patterned—through phase tracking to disrupt specific pathological neural activity—can achieve clinically useful symptom control offers a potentially highly selective form of electrical brain stimulation that can be extended to other disorders as underlying causal circuit mechanisms become clear.

## Supplementary Material

Supplementary Data

## Abbreviations

DBS: deep brain stimulation
TEED: total electrical energy delivered
