# Supplementary material for: Stimulating at the right time: phase-specific deep brain stimulation
Source: Brain. 2016 Dec 22;140(1):132–45. doi: 10.1093/brain/aww286 (PMC5226063; doi:10.1093/brain/aww286)
Supplement: Supplementary Data [file aww286_supp.pdf]

Supplementary Materials

Subject	Age	Gender	Time since symptom onset	Time since electrode implantation	Family history	Alcohol responsive
1	74	M	50 years	post-operative	Yes	N/A
2	76	M	18 years	0.5 years	Yes	No
3	73	M	55 years	5 years	Yes	N/A
4	64	M	50 years	4 years	Yes	Yes
5	74	F	All her life	11 months	Yes	Yes
6	67	F	53 years	4.5 years	Yes	Yes
7	79	M	12 years	post-operative	Yes	No
8	70	F	55 years	post-operative	Yes	No
9	77	M	30 years	3 years	Yes	No
10	67	M	5 years	3 years	N/A	N/A

Table S1. Patient details including time since tremor onset, electrode implantation date, family history of tremor related disorders and alcohol responsiveness of patient's tremor.

<table>
<tr><th>Case</th><th>Phase tracking stability</th><th></th></tr>
<tr><td>1</td><td>0.70</td><td rowspan="7">average for essential tremor patients: 0.76</td></tr>
<tr><td>2</td><td>0.29*</td></tr>
<tr><td>3</td><td>0.98</td></tr>
<tr><td>4R</td><td>0.97</td></tr>
<tr><td>4L</td><td>0.98</td></tr>
<tr><td>5</td><td>0.39</td></tr>
<tr><td>6</td><td>0.98</td></tr>
<tr><td>7</td><td>0.99</td><td rowspan="3">average for dystonic tremor patients: 0.71</td></tr>
<tr><td>8</td><td>0.54*</td></tr>
<tr><td>9</td><td>0.61</td></tr>
</table>

Table S2: Phase tracking stability. Using the instantaneous tremor phase derived from the band-pass filtered tremor signal, we determined the phase tracking efficacy of our algorithm. We computed the vector length of average tremor phase at which stimulation was delivered during a five second long stimulation block across all trials. If stimulation was delivered on average at the same tremor phase across all trials, then vector length would be 1, otherwise, if there was not phase consistency between trials, then vector length would be 0. For each trial, patients reassumed the tremor provoking posture. *These two cases did not show significant phase-amplitude effects.

Subject	Onset of Stimulation	At the end of phase specific stimulation
1	0.15 m/s^2<br>0.09 m/s^2	0.04 m/s^2<br>0.01 m/s^2
3	0.2 m/s^2	0.14 m/s^2
4R	0.11 m/s^2<br>0.08 m/s^2<br>0.19 m/s^2<br>0.17 m/s^2	0.16 m/s^2<br>0.11 m/s^2<br>0.07 m/s^2<br>0.08 m/s^2

Table S3. Tremor severity at the end of phase specific stimulation, when tremor severity was less than 0.2m/s^2 at the onset of stimulation. During prolonged phase specific stimulation, certain trials were excluded if tremor severity was less than 0.2 m/s^2 at the onset of stimulation: Subject 1: 2 out of 9 trials; Subject 3: 1 out of 10 trials; Subject 4R: 4 out of 7 trials.

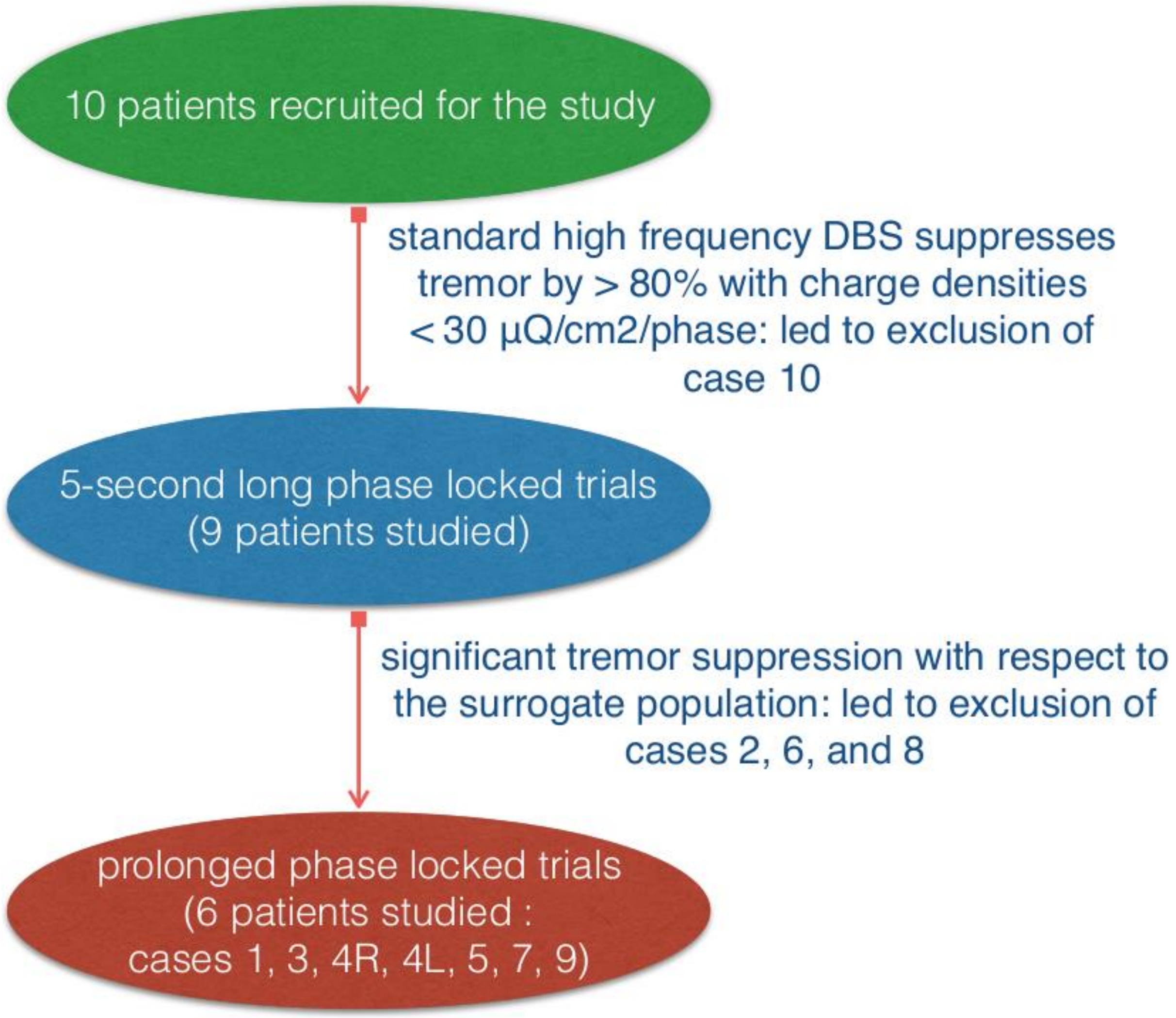

Fig. S1. Flowchart depicting the patient selection process

Stimulation Pattern

During phase specific DBS, a burst of high frequency pulses was delivered at each tremor cycle (Fig. S2). The intra-burst frequency was equivalent to the stimulation frequency used during conventional high frequency DBS (Table 1). For chronically implanted patients (cases 2 - 6, and 9), each burst of stimulation lasted for 35 ms (i.e. 4-6 pulses per tremor cycle), while for externalized patients (subjects 1, 7, and 8), 6 pulses were delivered per tremor cycle. The inter-burst frequency was defined according to the patient's tremor frequency.

The exact timing of stimulation was defined with respect to the last zero-crossing, derived from the band-pass filtered limb acceleration. For subjects 1 - 7, and 9, the stimulation time point (in seconds) from a zero-crossing was defined as

$$\mathrm{time_{burst}} = {\emptyset(1/\mathrm{f_{average\ tremor}})}/{360°}$$

Where ϕ stands for stimulation phase in degrees. Stimulation phases between 0°-180° were derived from the positive zero-crossing, while stimulation phases between 180°-360° were derived from the negative zero-crossing. For subjects 1 - 7, and 9, the absolute deviation from the reference tremor frequency was 0.48 Hz (taking into account the 25th and 75th percentiles of instantaneous tremor frequency). For subject 8, the tremor frequency from the previous tremor cycle was used to determine the precise time point for delivering stimulation at a particular phase ϕ (in degrees).

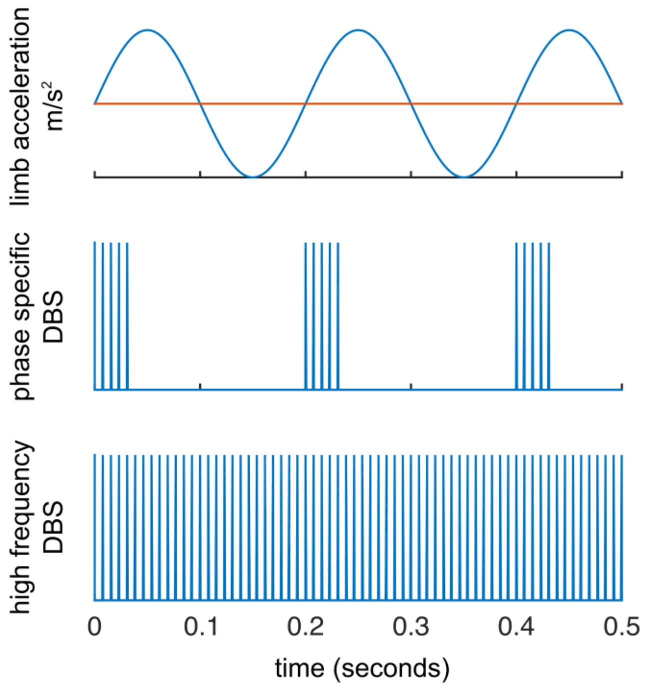

Fig S2. Stimulation pattern used during phase specific and high frequency DBS. During phase specific DBS, a burst of pulses was delivered at each tremor cycle. The intra burst frequency was the same as that used during conventional high frequency DBS (Table 1), while the inter burst frequency was determined according to patient's tremor frequency. The example depicts the stimulation pattern used when stimulation is delivered at 0° with respect to the band pass filtered hand acceleration.

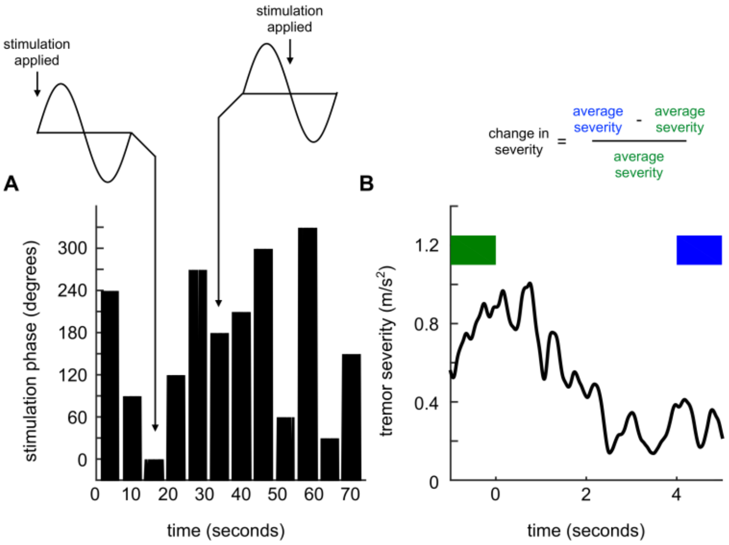

Fig. S3. Estimation of stimulation efficacy during randomized phased locked stimulation trials. (A) One trial of randomized phasic stimulation. Prior to stimulation onset, patients were asked to assume a tremor provoking position and to hold the position for 71 seconds. Stimulation phase was chosen from 0° to 330° in steps of 30° and stimulation was locked to a certain tremor phase for 5 seconds. Between trials, the order of stimulation phase was randomized. **(B)** Effect of a certain stimulation phase was assessed directly from the envelope of the accelerometer signal (m/s^2). The average change in tremor severity at the last second of stimulation (4 to 5 seconds - blue) with respect to average tremor severity prior to the onset of stimulation (-1 to 0 seconds green) is normalized by the average tremor severity prior to the onset of stimulation (illustration derived from subject 1).

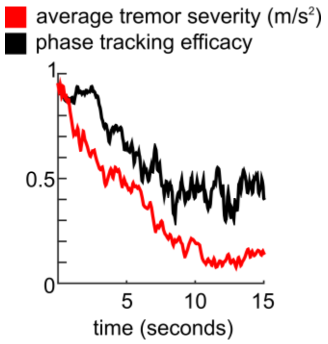

Fig. S4. The relationship between phase tracking stability and change in tremor severity with phase specific stimulation. Phase tracking stability decreased with reducing tremor severity during phase specific stimulation (illustration derived from subject 1). Phase tracking stability is estimated using a moving window phase synchrony index across 25 consecutive stimulation angles. Phase synchrony index (i.e. average vector length of 25 stimulation angles) of 1 indicates that stimulation angle was the same for the 25 consecutive stimulation pulses, while phase synchrony index of 0 indicates that stimulation angle was randomly selected from a uniform distribution.

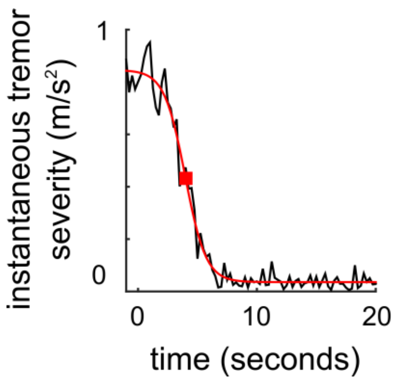

Fig. S5. Time course of tremor suppression. Black line shows the time course of tremor severity during a single trial of prolonged phase specific DBS. The red line shows the sigmoid fit while the red square depicts the time point when 50% of the maximum stimulation effect was reached for this trial. The maximum stimulation effect was 96% suppression (subject 1).

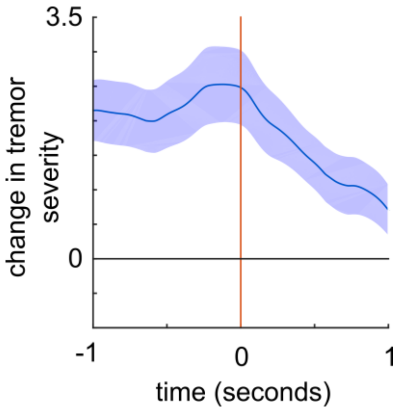

Fig. S6. Change in tremor severity aligned to termination of 5-second long phase specific stimulation trials in case 6. The reduction in tremor severity at the end of each stimulation block, coupled with the exclusively tremor amplifying effect of phase specific stimulation, is consistent with direct activation of the cortico-spinal tract. Solid blue line indicates the average change in tremor severity, while the shaded region indicates the SEM across all stimulation trials (N=108). All trials, regardless of stimulation phase, were used for this figure. Orange line indicates stimulation termination.

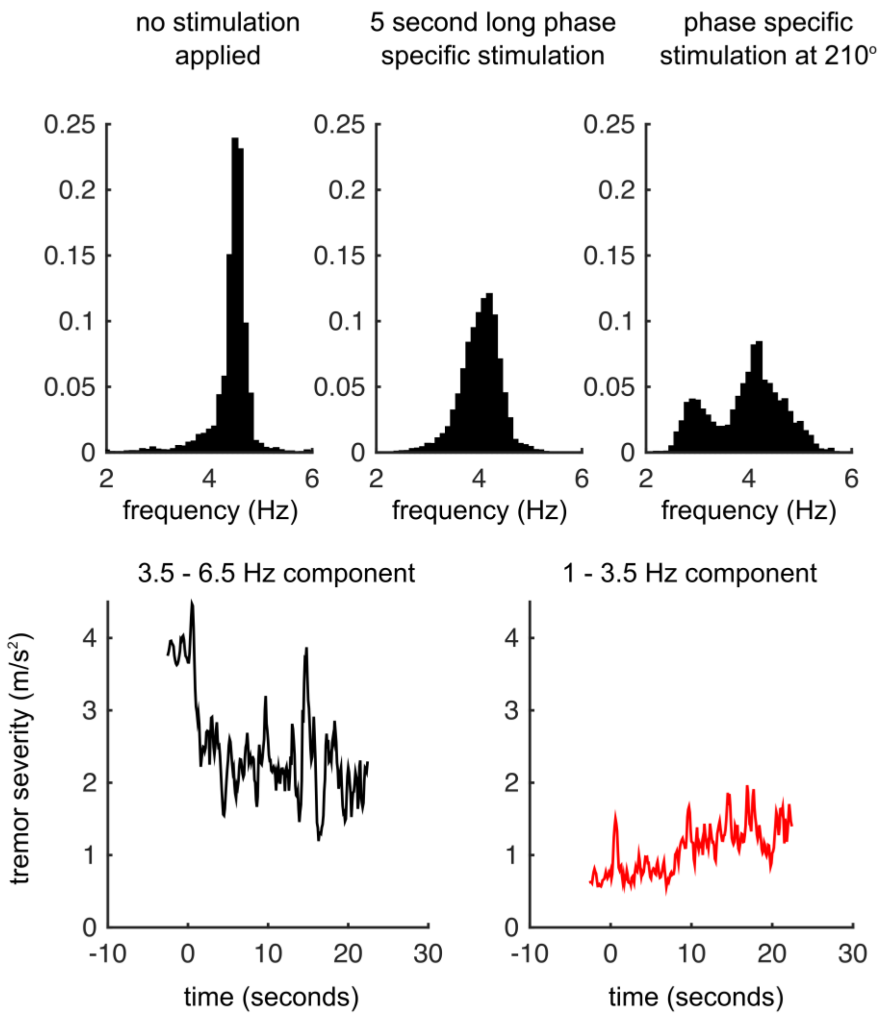

Fig. S7. Multiple independent tremor oscillators. For Case 5, during prolonged phase specific stimulation, as the dominant frequency component was suppressed, a new tremor oscillation emerged at a lower frequency, indicating that suppressing one tremor oscillator could facilitate emergence of an independent component.

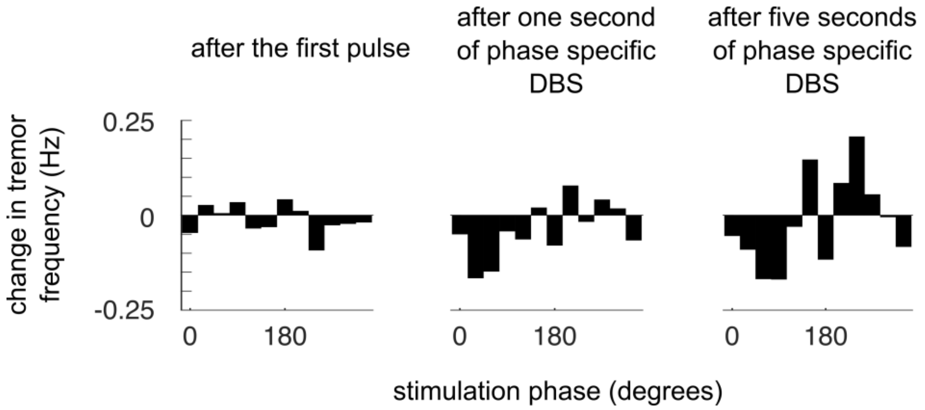

Fig S8. Changes in instantaneous tremor frequency during phase specific DBS of the ventrolateral thalamus. Following the first stimulation pulse phase locked to a certain tremor angle, the instantaneous tremor frequency did not change significantly depending on the stimulation phase (One way ANOVA p= 0.9661 df=11 number of trials=9). Similarly, there was not a main effect of stimulation phase on instantaneous tremor frequency after one-second of phase specific DBS (One way ANOVA p=0.3167 df=11 number of trials=9). However, stimulation phase locked to a certain tremor angle did increase or decrease the instantaneous tremor frequency, depending on the stimulation phase, after five seconds of phase specific DBS (One way ANOVA p= 0.0082). These changes in instantaneous tremor frequency are indicative of a Type II phase response curve, and highlight that stimulation effects require consistent phase locking before significant phase shifts can be induced within the cerebello-thalamo-cortical network. When a system described with a type II phase response curve is perturbed by stimulation, the period of the ongoing neural oscillation increases or decreases depending on the timing of stimulation with respect to the intrinsic oscillation. A reduction in the period of the ongoing neural oscillation (-Δ phase) corresponds to an increase in instantaneous frequency, while an increase in the period of the ongoing neural oscillation (+Δ phase) corresponds to a reduction in instantaneous frequency. The illustrated response curve is derived from case 1, and the most suppressive stimulation phase corresponded to the stimulation phase that maximally increased the instantaneous tremor frequency (240°).
